# Case report: de novo ANCA-associated vasculitis after kidney transplantation treated with rituximab and plasma exchange

**DOI:** 10.1186/s12882-018-1086-z

**Published:** 2018-10-19

**Authors:** Michael S. Sagmeister, Max Weiss, Peter Eichhorn, Antje Habicht, Rupert Habersetzer, Michael Fischereder, Ulf Schönermarck

**Affiliations:** 1 Nephrology Division, Medizinische Klinik und Poliklinik IV, Klinikum der Universität München - Campus Großhadern, Munich, Germany; 2 Department of Renal Medicine, University Hospitals Birmingham NHS Foundation Trust, Birmingham, UK; 3 Institute of Pathology, Ludwig-Maximilians-Universität, Munich, Germany; 4 Institute of Laboratory Medicine, Ludwig-Maximilians-Universität, Munich, Germany; 5 Centre for Transplantation, Klinikum der Universität München - Campus Großhadern, Munich, Germany; 6 KfH-Gesundheitszentrum Emmering / Dachau, Dachau, Germany

**Keywords:** ANCA, Vasculitis, Kidney transplantation, de novo, Rituximab, Case report

## Abstract

**Background:**

Anti-neutrophil cytoplasmic antibody (ANCA)-associated vasculitis causes end-stage renal failure in up to a third of cases even with treatment. The disease recurs occasionally after kidney transplantation, but new onset of ANCA-associated vasculitis after transplantation is highly unusual. The use of rituximab or plasmapheresis for de novo disease after transplantation has not previously been reported.

**Case presentation:**

Routine post-transplant follow-up for a 66-year old asymptomatic woman revealed a rise in creatinine from 1.8 to 2.6 mg/dl and increased proteinuria. She had received a cadaveric kidney transplant 20 months previously for end-stage autosomal dominant polycystic kidney disease. Renal allograft biopsy unexpectedly demonstrated pauci-immune glomerulonephritis with extracapillary proliferation and interstitial inflammation. Concurrent serum tested strongly positive for ANCA specific to proteinase 3 (PR3), but stored pre- and post-transplantation serum samples tested negative. These findings established a diagnosis of de novo ANCA-associated vasculitis in the renal allograft.

We started treatment with high-dose corticosteroid and rituximab. Despite this, serum creatinine continued to rise and glomerulonephritis remained active in a repeat biopsy. Escalation of the treatment with seven sessions of plasmapheresis led to a temporary improvement in creatinine. No further features of vasculitis emerged and PR3-ANCA titres declined. However, multiple infections complicated the recovery period and were associated with progressive loss of renal transplant function. Four months after the index presentation, transplant function became insufficient and dialysis was restarted.

**Conclusions:**

De novo ANCA-associated vasculitis after renal transplantation is exceptionally rare. It poses a significant risk to graft survival even in the context of intensified immunosuppression. Management relies on clinical evidence from populations with native renal function, yet post-transplant patients may be at increased risk of treatment-related adverse events. Precautions against these risks are crucial in the delivery of care.

## Background

Anti-neutrophil cytoplasmic antibody-associated vasculitis (AAV) is a rare autoimmune disorder leading to necrotising inflammation of small blood vessels and tissue damage [1]. It commonly affects the kidneys causing end-stage renal failure (ESRF) in 20–40% of cases [2]. Advancement in the management of AAV has been achieved through randomised controlled trials in populations with native renal function [3]. These have validated cyclophosphamide, and more recently rituximab, in combination with corticosteroids as effective treatments.

Less is known about AAV affecting renal transplants. Relapse of AAV in kidney transplants has been described for 42 cases as part of multiple small case series, not counting individual case reports [4]. Graft loss resulted in over a third despite intensified immunosuppression. Only two cases of de novo AAV after kidney transplantation have been described [5, 6]. Both occurred more than 10 years after transplantation, were treated with corticosteroid or corticosteroid plus cyclophosphamide and resulted in significant deterioration of graft function. We report a case of de novo AAV within 2 years of transplantation whose treatment included rituximab and plasmapheresis. We describe the presentation and course of AAV in this unusual context and discuss the evidence basis that informed our management.

## Case presentation

A 66-year old woman of Turkish descent attended our clinic in January 2015. She was asymptomatic at a routine follow-up 20 months after kidney transplantation and had an unremarkable physical examination. Of note, her creatinine had risen from 1.8 to 2.6 mg/dl since April 2014 and the urinary protein-creatinine-ratio had increased from 200 to 440 mg/g. Microhaematuria was absent at first, but became evident on repeat testing within 1 week (20 red cells per high-power field, no red cell casts).

Her background medical history consisted of coronary artery disease, hypertension, asymptomatic sinusitis and obesity. She had no known history of connective tissue or autoimmune disease. She had reached ESRF secondary to autosomal dominant polycystic kidney disease at the age of 58 years. After 7 years of haemodialysis, she received a deceased-donor kidney transplant without induction immunosuppression in May 2013 (baseline characteristics and human leucocyte antigen (HLA) genotyping of recipient and donor are shown in Table 1). An episode of asymptomatic cytomegalovirus (CMV) reactivation (1160 CMV copies / ml) 8 months after transplantation responded to valganciclovir and reduction of mycophenolate dose. At the index presentation, medications had been unchanged for more than 3 months. These were prednisolone 5 mg OD, cyclosporine A 50 mg BD, pantoprazole 20 mg OD, metoprolol 47.5 mg BD, doxazosin 4 mg BD, aspirin 100 mg OD, simvastatin 20 mg OD, allopurinol 150 mg OD and calcitriol 0.25 μg OD.

**Table 1:** Transplant donor and recipient characteristics, including genetic HLA types

	Donor	Recipient
Age (years)	73	65
Gender	male	female
CMV serostatus	positive	positive
MHC class I	A*02, 68	A*01, 32
	B*51,53	B*35, −
	C*04,14	C*04, −
MHC class II^a^	DRB1*13, 08	DRB1*11, 14
	DQB1*06, 04	DQB1*03, 05
Panel reactive antibody		0%
Cold ischaemia time (hours)	10,5	

^a^HLA-DP was not tested as part of the transplant matching process

**Fig. 1:**
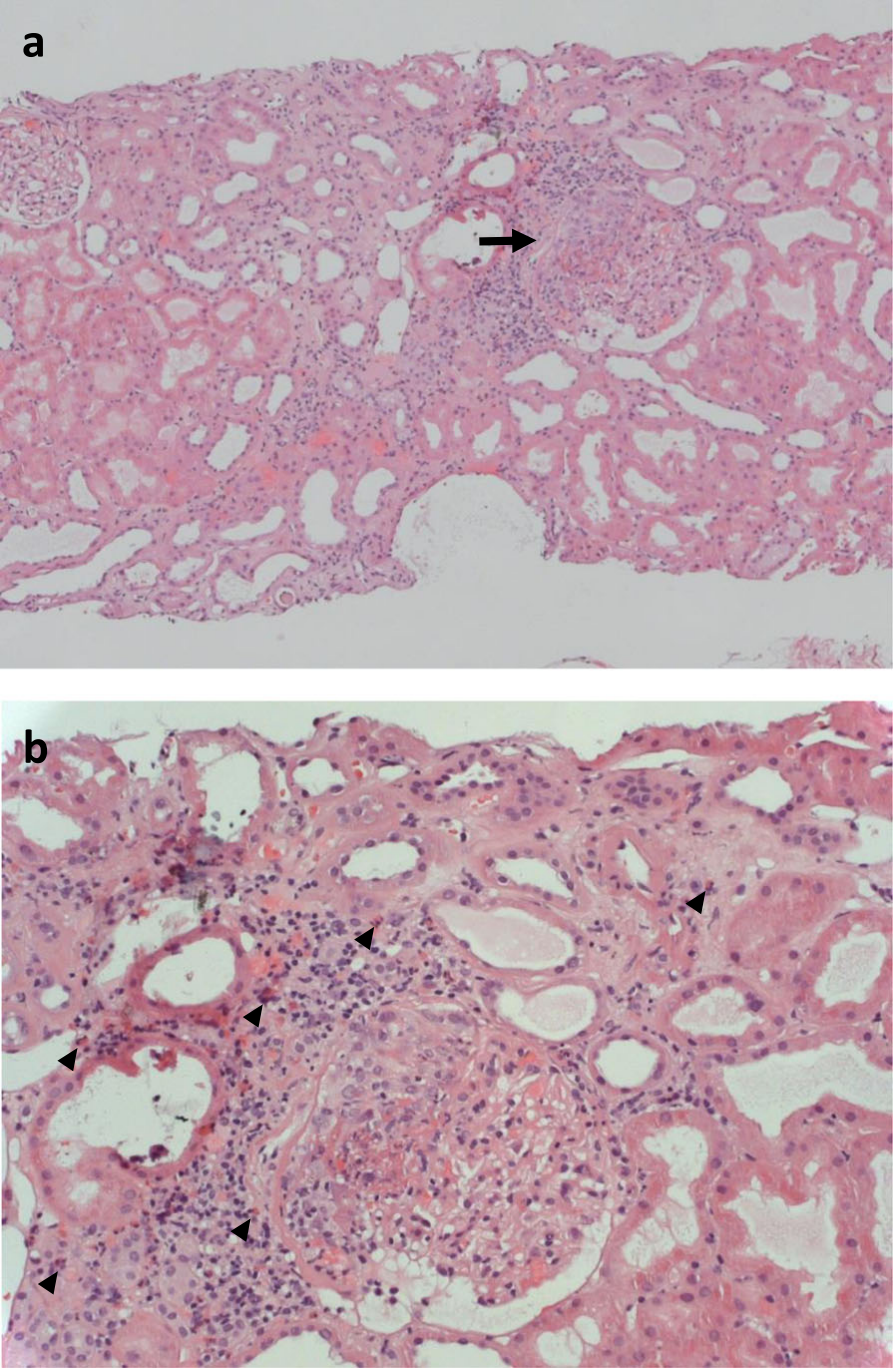
Light microscopy findings for the first biopsy of the transplant kidney. **a** Renal cortex with one normal glomerulus (top left corner) and one glomerulus exhibiting extracapillary proliferation, loss of integrity in Bowman’s capsule, fibrin deposition and focal necrosis (arrow; haematoxylin and eosin staining, original magnification × 100). **b** Detailed view of pathological glomerulus from above. There is also an interstitial inflammatory infiltrate including multiple eosinophilic granulocytes (arrowheads; original magnificent × 400)

A biopsy of the kidney transplant in response to the unexplained rise in creatinine showed eleven glomeruli, three of which were globally sclerotic. Unexpectedly, three of the remaining glomeruli demonstrated extracapillary proliferative changes, with crescent formation and necrosis in two (Fig. 1). Interstitial inflammation with eosinophilic cells and borderline changes suspicious for acute cellular rejection (Banff 3) were also seen. The lack of deposition of complement or immunoglobulins indicated a histological diagnosis of pauci-immune crescentic glomerulonephritis.

Autoimmune serology tested strongly positive for anti-neutrophil cytoplasmic antibodies (ANCA) with a typical cytoplasmic immunofluorescence pattern and specificity to proteinase 3 (PR3) (Euroimmun, Germany). ANCA specific to myeloperoxidase (MPO), anti-glomerular basement membrane antibodies, anti-nuclear antibodies and cryoglobulins were negative; complement and alpha-1-antitrypsin levels were normal. Donor-specific HLA antibodies were tested at time of biopsy and were negative. Retrospective analyses of frozen serum samples from 49 days prior and 36, 63, 96, 226 and 335 days post-transplantation were all negative for PR3-ANCA or MPO-ANCA. Lung involvement could be excluded with CT scan. A clinical review for vasculitis-typical features revealed that nasal crusting and bloody nasal discharge, the only extrarenal manifestations, had developed several months before the transplant biopsy. All of these findings supported the diagnosis of AAV and its specific subtype granulomatosis with polyangiitis.

We commenced treatment with intravenous prednisolone 250 mg for 3 days followed by rituximab 1 g and oral prednisolone 50 mg/day. Despite this, serum creatinine rose to 4.1 mg/dl over the next 2 weeks, prompting a second transplant biopsy 19 days after the first. The repeat biopsy contained 14 glomeruli, four of which were globally sclerotic. Three of the remaining glomeruli showed extracapillary proliferative changes and one of these exhibited necrosis. Minor features of ischaemia and mild interstitial inflammation were present, but no signs of rejection. Immunohistochemistry and electron microscopy remained consistent with pauci-immune glomerulonephritis.

**Fig. 2:**
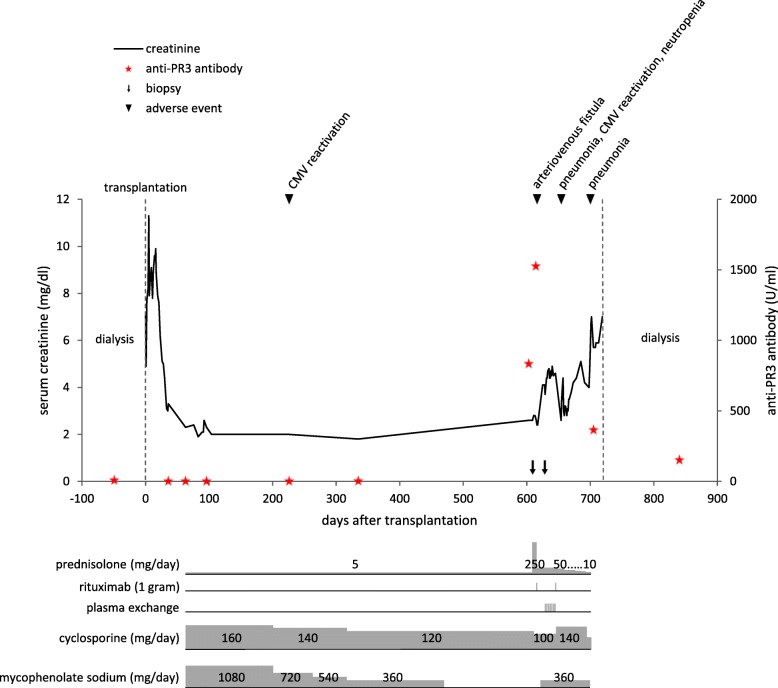
Overview of the patient’s clinical course and management

In view of the refractory disease course, we added seven sessions of plasma exchange. This was followed by a second administration of rituximab 1 g, a tapering course of oral prednisolone and antibiotic prophylaxis with co-trimoxazole. After 2 weeks, serum creatinine declined to 2.6 mg/dl, suggesting a partial recovery of renal graft function. The patient’s clinical course is summarised in Fig. 2.

A series of complications ensued. The patient developed corticosteroid-related diabetes mellitus, pneumonia with neutropenia and CMV reactivation in serum (586 copies / ml), requiring antimicrobial and antiviral therapy. Interventional occlusion of an arteriovenous fistula that had emerged after the first biopsy did not avert the rising trend in creatinine. A second infection episode occurred, fluid retention became problematic and creatinine rose to 7 mg/dl. Regular haemodialysis was recommenced from May 2015 onwards, 24 months after transplantation and 4 months after the initial presentation with rising creatinine and proteinuria. Transplant related immunosuppressive therapy with cyclosporine and mycophenolate was tapered and consecutively stopped while low-dose therapy with prednisolone for treatment of AAV was continued. The transplanted kidney was resected in September 2015 due to the new development of donor-specific HLA antibodies (anti-HLA-A2) and persistent haematuria. Histological examination found chronic and active antibody-mediated and cellular rejection, but no extracapillary proliferation. Rituximab was continued for maintenance therapy. PR3-ANCA remained positive, but declined steadily. There has been no further clinical vasculitis activity during follow-up for 2 years.

## Discussion and conclusions

We present a case of de novo AAV 20 months after kidney transplantation. The diagnosis of AAV is based on histological presence of pauci-immune crescentic glomerulonephritis and positive PR3-ANCA. No prior history of vasculitis symptoms and ANCA-negative tests on stored serum samples going back to the pre-transplant and immunosuppression-naïve phase support a de novo manifestation of the disease.

Our literature search found only two distinct cases of de novo AAV after kidney transplantation (13 and 14 years post-transplantation) [5, 6]. We suggest three explanations for the scarcity of this phenomenon. Firstly, it represents a rare disease in a small population at risk (incidence rate of AAV in the general population: 13 to 20 per million; prevalence of patients with kidney transplant in the UK: 462 per million) [7, 8]. Secondly, verification of AAV as de novo after transplantation is not always feasible. Thirdly, maintenance immunosuppression for prevention of transplant rejection is likely to protect against development of AAV, given that the relapse rate of AAV after renal transplantation is lower than in patients with native renal function or on dialysis [9]. Anti-rejection therapies downregulate T cells, which have been implicated in the pathophysiology of AAV. Immunosuppression in transplant patients is however best understood as threshold modulation for adaptive immune responses rather than their complete suppression, as illustrated by the occurrence of de novo donor-specific antibodies or immunological responses to vaccination [10, 11]. For these reasons, de novo AAV will remain exceptionally rare in kidney transplant patients, but close monitoring practises for this group will lead to detection of further cases.

The aetiology of AAV is not fully understood, but involves genetic as well as environmental factors. Genes from the major histocompatibility complex and other loci influence disease susceptibility and the clinical course [12, 13]. The HLA type DRB1*14, present in our patient, is linked with a moderate predisposition to AAV (odds ratio 1.91). Environmental risk factors may precipitate AAV, but antecedent infection, pertinent medications, silica exposure or heavy metal exposure were not present as far as we can tell [8, 14]. Reports about the deceased donor and the contra-lateral kidney recipient were unremarkable for vasculitis or autoimmune diseases. Our patient’s immunosuppression had been reduced in response to CMV reactivation and this may have diminished protection against AAV development. While ANCA are believed to emerge from pro-inflammatory milieus within tissues, we cannot tell if the interstitial inflammation and mild tubulitis seen in the initial biopsy are cause or effect in relation to AAV. To our knowledge, there were no infections, rheumatologic symptoms or new medications at the time that might point to an alternative cause for interstitial inflammation. As with nearly all cases of AAV, an explanation of the aetiology remains elusive.

For the first time, we report the use of rituximab for de novo AAV after kidney transplantation. Clinical evidence on the management of AAV in the post-transplant setting is scarce. In the two reported cases of de novo AAV, treatment with steroid and cyclophosphamide or steroid alone led to graft loss after 5 years or significantly impaired graft function at 6 months respectively [5, 6]. There are several retrospective case series on the management of disease relapses after transplantation, the largest comprising 13 patients [4, 15]. While older reports more commonly quote the use of cyclophosphamide, we found thirteen cases that were treated with rituximab [15–21]. Twelve of these responded to rituximab-based therapy, although publication bias may exaggerate this apparent efficacy. The strongest rationale for the use of rituximab comes from two randomised controlled trials in populations with native renal function which show equivalent efficacy and safety of rituximab compared to cyclophosphamide [22, 23]. The rare nature of post-transplant AAV means that clinicians have to rely on clinical evidence from related contexts like these trials to inform their management. We argue that rituximab can be considered as a therapeutic option. Even though kidney function could not be preserved declining PR3-ANCA levels and the lack of further vasculitic organ manifestations during follow-up demonstrate at least partial effectiveness in our case.

The initial lack of response to treatment brought additional complexity to our case. The rate of refractory AAV has been 2% to 5% in recent clinical trials for populations with native renal function [24]. We opted to escalate treatment with plasmapheresis. Although this improves renal survival without impact on overall survival for patients with severe renal impairment at presentation according to previous trial evidence [25], the final results of a recent large international trial are awaited to clarify the role of plasmapheresis as adjunctive therapy in first-line management (PEXIVAS; clinicaltrials.gov ID: NCT00987389). There is currently no consensus for the management of treatment-resistant AAV and alternative strategies are discussed in recent reviews [24, 26]. Further uncertainty concerns the maintenance immunosuppression in transplant patients with AAV. Commonly used anti-rejection agents are probably equivalent in prophylaxis of AAV relapses, but their handling during or after AAV (re-)induction therapy has received little consideration [9]. We re-introduced mycophenolate at a low dose, acknowledging also signs of mild cellular rejection in the first biopsy. These challenges from our case are infrequent but can be serious problems in AAV. Addressing them will require further research including high-quality retrospective studies.

Despite increased immunosuppressive therapy further decline of kidney function occurred as a consequence of vasculitic damage. However, the eventual loss of transplant function in our patient was partly due to treatment-related adverse events limiting further intensification of therapy. Their influence, particularly with respect to infections, on morbidity and mortality in AAV is well characterised [27, 28]. As observed in our case, the commonest type of infection is pneumonia, the risk is highest during the most intense phase of immunosuppression and infections are associated with leukopenia and renal impairment. Adverse event rates specifically for AAV therapy after transplantation are not available. A higher burden of co-morbidities, lower renal function, pre-existing immunosuppression and sensitisation of the immune system to graft rejection may predispose this cohort to complications. Rituximab and plasmapheresis have an expanding range of applications in the post-transplant setting and respective data on their safety profiles is emerging [29, 30]. Bearing in mind the devastating prognosis of untreated AAV, successful management depends on the right level of immunosuppression that will control vasculitis activity and limit adverse events. Achieving this balance is a key research area for AAV currently and is especially challenging for post-transplant patients. Close monitoring and careful precautions are therefore crucial to avert poor outcomes related to treatment.

De novo AAV after renal transplantation is exceptionally rare. There is insufficient data to elucidate possible differences in pathophysiology or prognosis of AAV in transplant kidneys compared to native kidneys. Active AAV in the renal allograft carries a significant risk of graft loss even in the context of intensified immunosuppression. Current management is informed by evidence from randomised controlled trials on patients with native renal function and case series on patients with AAV relapse after renal transplantation. Despite the availability of rituximab as a novel therapeutic option, treatment-related adverse events remain problematic.

## Abbreviations

AAV: Anti-neutrophil cytoplasmic antibody-associated vasculitis
ANCA: Anti-neutrophil cytoplasmic antibody
BD: Twice daily
CMV: Cytomegalovirus
ESRF: End-stage renal failure
HLA: Human leukocyte antigen
MHC: Major histocompatibility complex
MPO: Myeloperoxidase
OD: Once daily
PR3: Proteinase 3
